# Serum Fetuin-A Levels Related with Microalbuminuria in Diet-Induced Obese Rats

**DOI:** 10.1155/2013/795103

**Published:** 2012-12-31

**Authors:** Yanyan Li, Xiaodong Sun, Yerong Yu

**Affiliations:** Department of Endocrinology and Metabolism, West China Hospital, Sichuan University, Chengdu, Sichuan 610041, China

## Abstract

The aim of the study was to investigate the association between elevated serum fetuin-A and increased urine albumin excretion in obese rats, and whether increased urine albumin excretion was modified by improving hepatic steatosis and lipid metabolism disorder. Male Wistar rats 4 weeks in age were randomly divided into three groups and fed with normal chow (control group), high-fat chow (obesity group), or high-fat chow plus fenofibrate (fenofibrate group). After 24 weeks, both body weight and visceral fat/body weight ratio in obese rats were higher than in controls. A difference in serology markers and pathology associated with hepatic steatosis was also found among the three groups. Serum fetuin-A and the expression of NF-**κ**B in the liver were increased, while serum adiponectin was decreased in obese rats in comparison to controls (*P* < 0.01). Urinary albumin/creatinine ratio (ACR) was increased in the obesity group compared to controls (*P* < 0.01). The fenofibrate intervention reduced serum fetuin-A and NF-**κ**B expression in the liver and increased serum adiponectin compared to obese rats and was accompanied by decrease in ACR. A positive correlation was found between ACR and fetuin-A (*r* = 0.602, *P* < 0.01), and a negative correlation was found between ACR and adiponectin (*r* = −0.635, *P* < 0.01). We conclude that elevated fetuin-A levels are associated with microalbuminuria in obese rats, and abnormal albuminuria is reversible by improving hepatic steatosis.

## 1. Introduction 

In recent decades, changes in lifestyle and diet have led to an increased frequency in overweight and obese individuals. Obesity has become one of the most urgent public health problems and causes a major threat to human health worldwide. Obesity is associated with chronic diseases such as nonalcoholic fatty liver disease (NAFLD) and chronic kidney disease (CKD), among others. Growing recognition of the importance of obesity-related NAFLD and obesity-related CKD has provoked interest in the potential link between NAFLD and CKD. There is increasing evidence that NAFLD is associated with an increased prevalence and incidence of CKD. This association seems to be independent of obesity, hypertension, diabetes, and other possibly confounding factors [1, 2]. Moreover, prediabetic and newly diagnosed diabetic patients with NAFLD had higher prevalence rates of microalbuminuria than those without NAFLD [3]. However, the mechanistic relationship between NAFLD and CKD remains unknown. 

Fetuin-A is an abundant serum protein, that is, predominantly synthesized and secreted by the liver. Higher serum fetuin-A levels have been found in diet-induced obesity, NAFLD and metabolic syndrome (MetS) [4–6]. Stefan and Haring reported that fatty liver might play an important role in the pathogenesis of the metabolic diseases type-2 diabetes mellitus (T2DM) and cardiovascular disease, and fetuin-A is directly involved in the pathogenesis of these diseases. However, recent novel findings revealed that under certain conditions fatty liver is not associated with metabolic disorders; the terms “metabolically benign” has been used to describe this state [7]. Adiponectin, a plasma protein primarily secreted by adipose tissue, was found to be decreased in NAFLD [8]. Both human fetuin-A and adiponectin genes reside on chromosome 3q27, which has been mapped as a T2DM and MetS susceptibility locus [9, 10]. Hennige and colleagues demonstrated that the liver-derived fetuin-A induced low-grade inflammation and repressed adiponectin production in animals and humans [11]. Moreover, adiponectin is a key regulator of albuminuria and is inversely related to albuminuria [12]. Low-grade inflammation, one of the characteristics of chronic kidney disease (CKD), is also associated with albuminuria. From this information, we hypothesized that high serum fetuin-A levels may be associated with increased urine albumin excretion. One study reported that serum fetuin-A level was elevated in subjects with fat accumulation in the liver, and a decrease in liver fat was accompanied by a decrease in serum fetuin-A concentration [13]. In addition, elevated fetuin-A level in obesity is significantly reduced during exercise- and diet-induced weight loss [4]. However, it is not known whether improving lipid metabolism disorder is associated with ameliorating elevated fetuin-A level or reversing increased urine albumin excretion.

Therefore, this study investigated whether high circulating fetuin-A is associated with increased urine albumin excretion in diet-induced obese rats and examined the potential mechanism associating fetuin-A with microalbuminuria. We also studied whether the increased urine albumin excretion induced by higher serum fetuin-A levels was modified by improving hepatic steatosis and lipid metabolism disorder. 

## 2. Methods 

### 2.1. Animal Studies

4-week-old male Wistar rats were purchased from the experimental animal center of west China, Sichuan University, China. The animal protocol was approved by the local ethics committee for animal studies and animals were handled according to “Principles of laboratory animal care”. All rats were housed under controlled temperature (20 ± 2°C), humidity (50%), and light conditions (12-h light-dark cycle), and water was freely available. The rats were randomly divided into three groups: the control group (*n* = 8) was fed with normal chow (320 kcal/100 g/day), the obesity group (*n* = 8) was fed with high fat chow (586 kcal/100 g/day) and the fenofibrate group (*n* = 8) was fed with high fat chow plus fenofibrate (100 mg/kg/d). At the end of 24 weeks the rats were sacrificed and the liver was harvested. Liver lobes were weighed and processed for liver triglycerides (LTG), protein, and histological analysis. The blood was collected and serum samples were stored and kept frozen at −80°C for analyzing the levels of alanine aminotransferase (ALT), aspartate aminotransferase (AST), free fatty acids (FFAs), fetuin-A, and adiponectin. Abdominal visceral fat (including perirenal fat, epididymal fat, and mesenteric fat) was collected and weighted.

### 2.2. Blood Measurements

Plasma glucose was measured by the glucose oxidase method. Serum insulin was assayed by radioimmunoassay (Linco Research, Inc., St Louis, MI, USA). Serum ALT and AST levels were measured by automatic biochemical analysis meter (Beckman, USA). Serum FFA concentrations were assayed by the colorimetric method with serum FFAs kit (E1001, Applygen Technologies Inc., Beijing, China). Serum fetuin-A and adiponectin levels were measured by an ELISA technique using the rat fetuin-A and adiponectin kits (E90178Ra and E90605Ra, resp., Uscn Life Science Inc., China), according to the manufacturer-supplied instructions. 

### 2.3. Analysis of Liver Triglycerides (LTG)

Frozen liver tissues were used for analyzing LTG using the tissue TG kit (E1013, Applygen Technologies Inc., Beijing, China). The liver was weighted and put into a manual glass homogenizer with lysis buffer (20 uL/mg), which was heated and then centrifuged to obtain supernatant for future enzymatic assays, according to the manufacturer-supplied instructions. 

### 2.4. Liver and Kidney Histological Examination

Hepatic and renal cortex tissues were excised from each rat, fixed in 10% formalin, and embedded in paraffin wax. Paraffin-embedded tissues were sectioned and stained with hematoxylin-eosin (H&E).

### 2.5. Liver NF-*κ*B p65 Immunohistochemical Staining

Sections of formalin-fixed, paraffin-embedded tissue were stained by means of an anti-rabbit SP kit (SP-9001, Zhong Shan Inc., Beijing, China). The specimens were incubated with rabbit anti-rat polyclonal NF-*κ*B (p65) antibody (1 : 200 dilution; sc-101748, Santa Cruz Biotechnology, Inc.) overnight at 4°C. The procedure was performed essentially as described [14]. Semiquantitative analysis of NF-*κ*B p65 expression in the liver used image analysis techniques (Image-Pro Plus software 6.0) to determine the gray scale value.

### 2.6. Measurement of Urine Albumin Excretion

Urine was collected in a metabolic cage at the end of 24 weeks. Urinary albumin was measured using a radioimmunoassay kit (China Institute of Atomic Energy, Beijing, China). Urinary creatinine concentration was measured by the Jaffe method. Urine albumin excretion was defined as urine albumin-to-creatinine ratio (ACR). 

### 2.7. Statistical Analysis

Statistical analysis was performed by the statistical software package SPSS 17.0 for Microsoft windows (SPSS Inc). Data were expressed as means ± standard deviation. For three group comparisons, data were analyzed by ANOVA. Statistical comparison among two groups was performed with the student's  *t*-test. Pearson correlation coefficient “*r*” was used to measure the relationship between two variables.  *P*  values less than 0.05 were considered statistically significant. 

## 3. Results 

Body weight increased 32.8% in obese rats in comparison to controls, and visceral fat increased 3.67 times in the obesity group compared to the control group. Compared with controls, obese rats had significantly higher visceral fat and weight (*P* < 0.01). In the fenofibrate group, body weight was duplicated (with some slight differences) compared to the obesity group, but visceral fat decreased 25.7% in the fenofibrate group when compared to the obesity group; visceral fat and weight were significantly decreased in the fenofibrate group in comparison to the obesity group (Table 1). 

There was a significantly increased level of serum insulin, free fatty acids (FFAs), ALT, AST, and LTG in obese rats compared to controls (*P* < 0.05, Table 2). A significant decrease in most measurements was detected when fenofibrate was compared to the obesity group (*P* < 0.05), except for LTG content (Table 2). Although the LTG content was apparently decreased in the fenofibrate group, no significant difference was found between the fenofibrate and obesity groups. The data show that the degree of variability was high in obesity group, which might cause the above phenomenon. 

**Figure 1:**
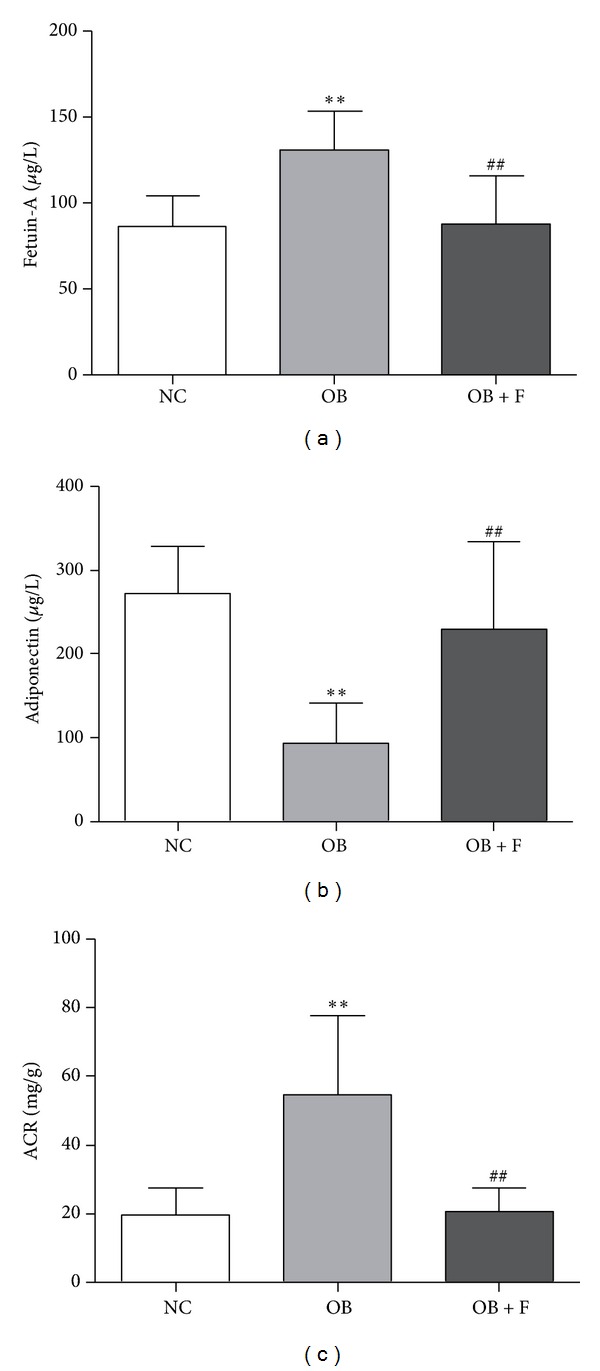
Representative parameters of rats in three groups: levels of fetuin-A (a), adiponectin (b), and ACR (c) in each group. Data was represented by means ± SD (*n* = 8). **P < 0.01 versus control group; ^##^ *P* < 0.01 versus obesity group; (NC = control group, OB = obesity group, OB + F = fenofribate group).

There was a significant increase in serum fetuin-A levels in obese rats compared to controls (130.88 ± 22.41 versus 86.24 ± 17.97 *μ*g/L, *P* = 0.001). A significant decrease was detected in the fenofibrate group when compared to the obesity group (87.94 ± 27.7 versus 130.88 ± 22.41 *μ*g/L, *P* < 0.01). Serum adiponectin levels were significantly reduced in obese rats in comparison to controls (93.69 ± 47.45 versus 272.27 ± 56.71 *μ*g/L, *P* < 0.001). A significant increase was found when the fenofibrate group was compared to the obesity group (228.70 ± 104.4 versus 93.69 ± 47.45 *μ*g/L, *P* < 0.01). Urinary albumin/creatinine ratio (ACR) was significantly increased in obese rats accompanied by higher fetuin-A levels and lower adiponectin levels compared to controls (54.47 ± 23.26 versus 19.62 ± 7.81 mg/g, *P* < 0.01). The decrease was significant when comparing the fenofibrate group to the obesity group (20.68 ± 6.67 versus 54.47 ± 23.26 mg/g, *P* < 0.01). Data are shown as mean ± SD (Figure 1).

**Figure 2:**
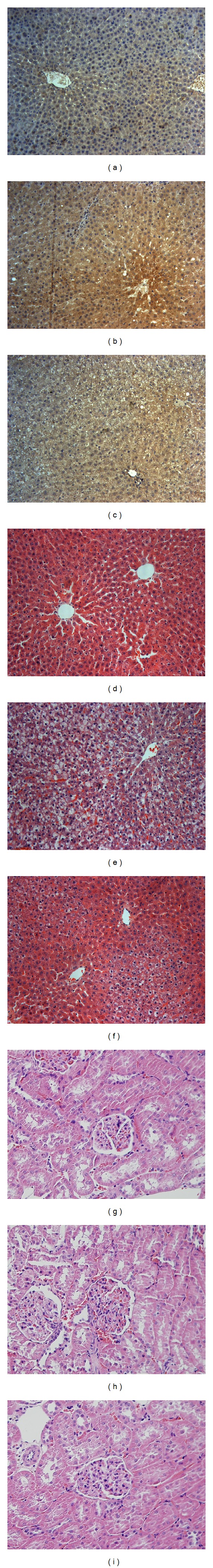
Immunohistochemical staining for NF-*κ*B p65 in the liver in control group (a), obesity group (b), and fenofibrate group (c), respectively. Liver H&E stain from control group (d), obesity group (e), and fenofribate group (f), respectively. Kidney H&E stain from control group (g), obesity group (h), and fenofibrate group (i), respectively. Original magnification ×200; (NC = control group, OB = obesity group, and OB + F = fenofribate group).

Positive NF-*κ*B staining was located in the cytoplasm or nucleus of the liver cells. The NF-*κ*B expression in the liver was apparently increased in the obese rats (Figure 2(b)) while little NF-*κ*B expression in the liver was observed in normal rats (Figure 2(a)). After fenofibrate intervention, the increase in NF-*κ*B expression observed in the liver of obese rats was suppressed (Figure 2(c)). Semiquantitative analysis of NF-*κ*B expression in the liver by image analysis techniques showed that there was a 26.3% increase in obese rats compared to controls (0.385 ± 0.031 versus 0.306 ± 0.028, *P* = 0.001) and an 18.9% decrease in the fenofibrate group compared to the obesity group (0.313 ± 0.045 versus 0.385 ± 0.031, *P* < 0.05). HE-stained liver from diet-induced obese rats revealed a significantly increased steatosis when compared to controls, which was significantly improved after fenofibrate intervention (Figures 2(d)–2(f)). HE-stained kidney revealed marked glomerular enlargement and increased interstitial inflammatory cells (Figures 2(g)–2(i)). However, lowering circulating and renal FFA levels by fenofibrate intervention reduced the glomerular hypertrophy.

**Figure 3:**
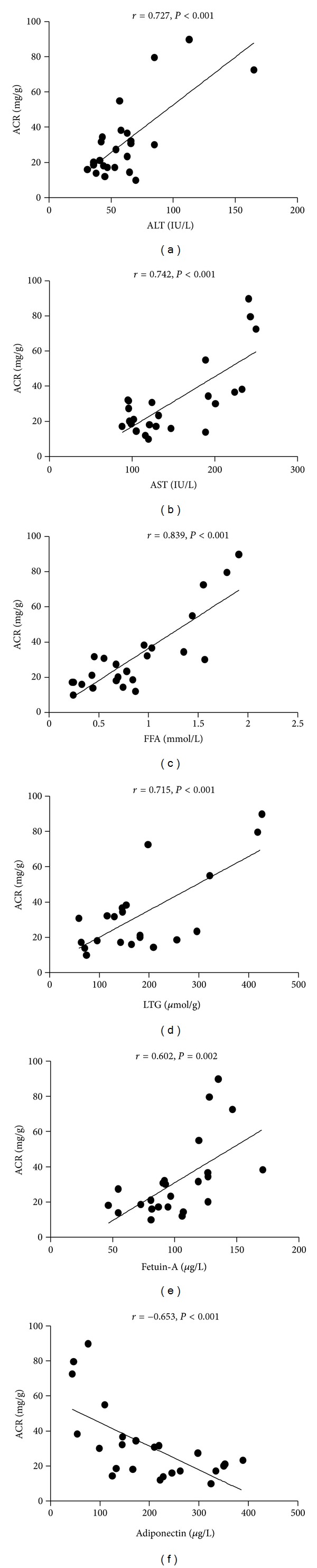
Correlation of serum ALT and AST, serum FFAs, LTG, serum fetuin-A, and adiponectin with the level of ACR; data are from three groups.

Serum FFA concentration and LTG levels were increased in diet-induced obese rats and accompanied by higher serum fetuin-A levels and higher ACR. However, after fenofibrate intervention, lipid metabolism was improved, and simultaneously serum fetuin-A levels were significantly decreased and urine albumin secretion was significantly reduced. A significant positive correlation was found between serum fetuin-A levels and ACR (*r* = 0.602, *P* = 0.002). Furthermore, a significant positive correlation was detected between ACR and each of these proteins: serum FFAs, serum ALT, serum AST, and LTG (*r* = 0.839, *P* < 0.001; *r* = 0.727, *P* < 0.001; *r* = 0.742, *P* < 0.001; *r* = 0.715, *P* < 0.001, resp.). In addition, there was a significant negative correlation between serum adiponectin level and ACR (*r* = −0.653, *P* = 0.001; Figure 3).

**Figure 4:**
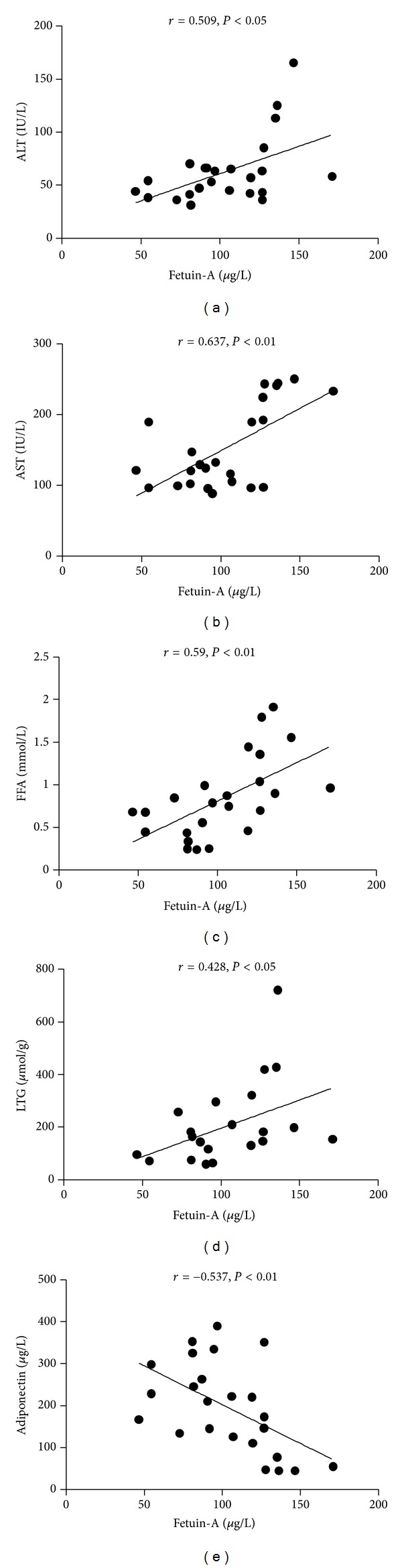
Correlation of serum ALT and AST, serum FFAs, LTG, and serum adiponectin with the level of serum fetuin-A; data are from three groups.

In addition, there was a significant positive correlation between serum fetuin-A and each of these proteins: serum ALT, AST levels, serum FFAs, and LTG content (*r* = 0.509, *P* < 0.05; *r* = 0.637, *P* < 0.01; *r* = 0.590, *P* < 0.01; *r* = 0.428, *P* < 0.05, resp.). A strong inverse correlation was found between serum fetuin-A and serum adiponectin (*r* = −0.537, *P* < 0.01; Figure 4).

We divided all parameters into tertiles based on ACR: lower, middle, and upper tertiles. Increasing tertiles of ACR were associated with increasing levels of fetuin-A, adiponectin, and NF-*κ*B (Table 3). In addition, there was an obvious increase in serum ALT, AST, and FFAs with increasing tertiles of ACR (Table 3). Although there was no significant difference in LTG content, an apparent increase was found accompanied by increasing tertiles of ACR.

## 4. Discussion

Chronic kidney disease (CKD) is defined by the presence of a marker of kidney damage, such as proteinuria or a decreased glomerular filtration rate for three or more months [2]. CKD has emerged as a growing public health problem worldwide. Microalbuminuria is an early marker of chronic kidney injury, and several cross-sectional studies have noted an association between metabolic syndrome and microalbuminuria in individuals with and without diabetes [15–17]. Ix et al. has documented that higher fetuin-A human concentrations are strongly associated with MetS and an atherogenic lipid profile [6]. Increasing quartiles of fetuin-A are linearly associated with the number of MetS components and increased serum triglyceride concentrations [6]. Additionally, fetuin-A levels strongly, and independently from other important parameters were found to be associated with hyperglycemia [18]. Fetuin-A has been studied as an important circulating inhibitor of ectopic calcium deposition in the renal field, and fetuin-A deficiency in humans was associated with vascular calcification and mortality in patients on hemodialysis [19]. Fetuin-A might, however, have other functions; fetuin-A is an important promoter of insulin resistance and exerts proinflammation effects. This led us to investigate whether higher serum fetuin-A is associated with abnormal albuminuria. 

In the current study, serum fetuin-A levels and microalbuminuria were measured in rats of three different intervention groups to clarify their potential relationship. We are able to show that there was a strong, positive correlation between serum fetuin-A levels and abnormal albuminuria, as reported previously by Li et al. [20]. The results showed a significant increase in serum fetuin-A levels in obese rats compared to controls. Our study proved that there is a significant positive correlation between serum fetuin-A level and serum ALT, AST level, serum FFA concentration, and LTG content. These results are in accordance with the previous studies that serum fetuin-A levels are elevated in NAFLD [4, 5, 21]. Yilmaz et al. found that serum fetuin-A levels are significantly higher in patients with biopsy-proven NAFLD and may serve as a biochemical marker of fibrosis in NAFLD patients [21]. However, after diet- and exercise- or surgery-induced weight loss, the homeostatic model assessment (HOMA) index and fetuin-A decreased, and a significant decrease in the prevalence of NAFLD was found [4, 22]. In addition, a cross-sectional and longitudinal study reported that serum fetuin-A levels were elevated in subjected with fat accumulation in the liver, and a decrease in liver fat was accompanied by a decrease in serum fetuin-A concentrations [13]. These findings raise the possibility that fetuin-A may be a new promising link between obesity and its comorbidities. Simultaneously, a significant increase was detected in ACR in obese rats compared to controls. This increase reflects renal impairment and suggests the presence of CKD in obese rats with higher serum fetuin-A levels. A number of studies have shown a positive correlation between obesity and microalbuminuria [23, 24], and that obesity is an independent risk factor for the development of microalbuminuria [25], which is also associated with the MetS and its different components. The prevalence of NAFLD has been estimated to be approximate 90% in obesity [26]; both NAFLD and dyslipidemia contribute to the rate of progression of renal disease [1, 24]. Therefore, higher serum fetuin-A is a potential risk factor in diet-induced obese rats and contributes to the prevalence of abnormal albuminuria. 

The previous studies have reported that a negative correlation was found between adiponectin and microalbuminuria in obese patients [12, 23]. Fetuin-A is deemed to repress the production of adiponectin [11]. Based on these findings, we further investigated the association of fetuin-A with adiponectin to clarify the potential mechanism that fetuin-A induced the increased urine albumin excretion. We found that serum adiponectin levels were significantly decreased in obese rats in comparison to controls, and our results revealed a strong inverse correlation between serum fetuin-A and serum adiponectin, and a significant inverse correlation between ACR and serum adiponectin. Adiponectin, an adipocyte-derived hormone, has anti-inflammatory properties by modulating multiple signaling pathways and exerts largely beneficial effects to improve insulin sensitivity. The decreased level of serum adiponectin represents an independent risk factor for NAFLD [27]. It is well known that chronic low-grade inflammation is a common feature of progression of NAFLD. The results of the present study are in accordance with previous studies [28, 29], showing that there was a higher levels of an inflammatory marker, as evidenced by the significant increase in liver NF-*κ*B expression, in obese rats compared to controls. These results are in accordance with a report that FFAs enhanced fetuin-A secretion commensurate with over-expression and activity of NF-*κ*B [30]. Another study demonstrated that fetuin-A acted as an endogenous ligand of TLR4, played a key role in FFA-induced proinflammatory cytokine expression, and promoted lipid-induced insulin resistance [31]. Moreover, the fatty liver itself can exacerbate insulin resistance, thus increasing the risk of renal impairment [32], and several proinflammatory factors from steatosis and inflamed liver can further exacerbate kidney dysfunction [33]. These findings are in accordance with the current study showing that increasing tertiles of ACR were related with a gradual increase in liver NF-*κ*B expression. These findings provide evidence that higher fetuin-A levels induce increased urine albumin excretion by a mechanism related with repressing adiponectin and triggering inflammation. 

Several studies have demonstrated that lifestyle intervention or surgery-induced weight loss leads to a significant decrease of fetuin-A [4, 22]. In a longitudinal study, a large decrease in liver fat induced by lifestyle intervention was paralleled by a decrease in serum fetuin-A levels [13]. Moreover, Ix et al. found a particularly strong association of human fetuin-A with an atherogenic lipid profile among the components of MetS. However, whether reduced FFA levels could reverse the elevated serum fetuin-A remain unclear. It is known that higher serum FFAs contribute to lipid ectopic deposition in liver, and our findings show that there was a significant increase in FFA concentration and LTG content in obese rats compared to controls. In addition, our study demonstrated that fenofibrate alleviated hepatic steatosis by regulating lipid metabolism (reduced serum FFAs and LTG content); the results also revealed a significant decrease in serum fetuin-A levels, which extends previous findings of decreased fetuin-A levels with improvement of NAFLD after a short-term lifestyle intervention [13]. These findings reflect the reversibility of the increased fetuin-A concentrations. However, in our present study we found that there was no significant difference of LTG levels between the obesity and fenofibrate groups, and that not every LTG level was significantly elevated in the obesity group. This condition indicated that in diet-induced obese rats, fatty liver stayed at a certain stage, as reported by the previous review [7], which is associated with a metabolically benign state. Interestingl, a significant increase was detected in serum adiponectin levels with fenofibrate treatment in comparison to the obesity group, which is in accordance with the previous study that adiponectin was increased after short-term fenofibrate therapy [13, 34]. Moreover, fenofibrate alleviated hepatic steatosis and decreased serum fetuin-A levels accompanied by improvement in hepatic chronic low-grade inflammation. As the results revealed, a significant decrease was detected in liver NF-*κ*B expression with fenofibrate compared to the obesity group. The steatotic and inflamed liver was alleviated after the fenofibrate intervention in the wake of a reduction in ACR. Therefore, the increased urine albumin excretion is reversible by improving hepatic steatosis. 

## 5. Conclusions

The results of the present study indicates that in diet-induced obese rats higher serum fetuin-A levels represent a novel risk factor for the presence of microalbuminuria, and the mechanism may be related with repressing adiponectin production and triggering of chronic low-grade inflammation. Abnormal albuminuria induced by increased fetuin-A levels is reversible by improving hepatic steatosis. Therefore, attention should be paid to obesity or NAFLD with early kidney lesions, and the use of therapeutic agents or lifestyle intervention could be used to prevent NAFLD patients from developing microalbuminuria, thus delaying further renal impairment. 

## Figures and Tables

**Table 1:** Biometric parameters of rats in the studied groups (*n* = 8).

Group	Body weight (g)	Visceral fat (g)	Visceral fat/weight (10^−3^)
Control group	521.3 ± 35.2	14.93 ± 1.68	28.58 ± 1.86
Obesity group	692.3 ± 93.9**	54.90 ± 14.18**	73.85 ± 18.76**
Fenofibrate group	657.4 ± 56.9**	40.78 ± 5.31^##∗∗^	55.71 ± 8.47^##∗∗^

Data are shown as mean ± SD. **P < 0.01 versus control group. ^##^ *P* < 0.01 versus obesity group.

**Table 2:** Blood parameters of rats in three groups (*n* = 8).

Group	FBG (mmol/L)	FINS (mIU/L)	ALT (IU/L)	AST (IU/L)	FFAs (mmol/L)	LTG (*μ*mol/g)
Control group	5.7 ± 0.4	0.52 ± 0.19	48.5 ± 13.7	124 ± 32.4	0.37 ± 0.12	110.9 ± 49.46
Obesity group	6.0 ± 0.4	1.62 ± 0.24*	83.6 ± 39.5*	222 ± 24.3*	1.45 ± 0.33*	258.8 ± 127.6*
Fenofibrate group	5.9 ± 0.5	0.89 ± 0.24^#∗^	51.5 ± 12.6^#^	108 ± 13.8^#^	0.78 ± 0.11^#∗^	192.4 ± 77.9*

Data are shown as mean ± SD. *P < 0.05 versus control group; ^#^ *P* < 0.05 versus obesity group. FBG: fasting blood glucose, FINS: fasting insulin, ALT: Alanine aminotransferase, AST: Aspartate aminotransferase, FFAs: free fatty acid, LTG: liver triglyceride.

**Table 3:** Parameters of rats by ACR tertiles.

	Lower tertile 14.7 ± 2.9 (mg/g)	Middle tertile 25.3 ± 5.2 (mg/g)	Upper tertile 54.7 ± 22.9 (mg/g)	*P *
Fetuin-A (*μ*g/L)	82.4 ± 22.1	91.9 ± 23.6	130.8 ± 22.6	0.001
ALT (IU/L)	49.1 ± 13.1	52.9 ± 17.5	81.3 ± 40.0	<0.05
AST (IU/L)	126.9 ± 30.4	118.4 ± 36.2	208.4 ± 51.2	<0.001
FFA (mmol/L)	0.47 ± 0.25	0.75 ± 0.36	1.38 ± 0.37	<0.001
LTG (*μ*mol/g)	117.3 ± 55.7	184.1 ± 85.1	240.9 ± 128.5	0.075
Adiponectin (*μ*g/L)	238.7 ± 71.4	256.6 ± 107.6	99.4 ± 50.8	0.001
NF-*κ*B	0.303 ± 0.046	0.314 ± 0.026	0.385 ± 0.031	<0.05

Data are shown as mean ± SD. Data was analyzed by ANOVA. ALT: Alanine aminotransferase, AST: Aspartate aminotransferase, FFA: free fatty acid, LTG: liver triglyceride.

## Abbreviations

ACR: Urinary albumin/creatinine ratio
ALT: Alanine aminotransferase
AST: Aspartate aminotransferase
CKD: Chronic kidney disease
FFAs: Free fatty acid
IR: Insulin resistance
LTG: Liver triglyceride
MAU: Microalbuminuria
MetS: Metabolic syndrome
NAFLD: Non-alcoholic fatty liver disease
T2DM: Type 2 diabetes mellitus.
